# IMRC-Exo mitigates *Deinagkistrodon acutus* venom-induced limb injury in rabbits by inhibiting GSDME-dependent pyroptosis

**DOI:** 10.1590/1678-9199-JVATITD-2025-0009

**Published:** 2025-09-05

**Authors:** Haohao Wu, Lutao Xie, Wang Du, Linjie Lai, Peixin Shangguan, Xingzhen Wu, Jiefeng Xu, Pin Lan

**Affiliations:** 1 Department of Emergency, The Fifth Affiliated Hospital of Wenzhou Medical University, Lishui Central Hospital, Lishui, Zhejiang, China.; 2 Department of Emergency Medicine, The Second Affiliated Hospital of Zhejiang University School of Medicine, Hangzhou, Zhejiang, China.

**Keywords:** Deinagkistrodon acutus, Snakebite, Snake venom, Immune and matrix-regulatory cell-derived exosomes, Pyroptosis, Gasdermin E

## Abstract

**Background:**

Inflammation plays a critical role in the pathogenesis of limb injury caused by *Deinagkistrodon acutus* snakebite. Investigating its regulatory mechanisms and intervention strategies may help identify effective treatments. Recent studies have shown that pyroptosis exacerbates organ damage by amplifying inflammatory responses. Additionally, immune and matrix-regulatory cells (IMRC), a novel type of mesenchymal stem cell, and their exosomes (Exo) have demonstrated potential in mitigating inflammation-mediated injury by suppressing pyroptosis. This study aimed to evaluate whether IMRC-Exo could alleviate *D. acutus* venom-induced limb injury in rabbits by suppressing pyroptosis, thereby attenuating the associated inflammatory response.

**Methods:**

Eighteen healthy male New Zealand white rabbits were randomly assigned to Sham, Model, and IMRC-Exo groups. The Model group was established by intramuscular injection of *D. acutus* venom (1.5 mg/kg), followed by intravenous snake antivenom (80 U/kg) after 2 hours. The IMRC-Exo group received IMRC-Exo (7.5 × 10^10^ particles) post-modeling. Within 24 hours, left thigh circumference, serum creatine kinase (CK), and myoglobin (Mb) were assessed. Muscle tissues were collected for histopathology, apoptosis analysis, inflammatory cytokine quantification [high-mobility group box 1 (HMGB1), IL-1β, IL-18], and pyroptosis-related protein detection [caspase-3, cleaved caspase-3, gasdermin E (GSDME), N-terminal GSDME (N-GSDME)].

**Results:**

Compared to Sham, venom injection significantly increased thigh circumference, CK, Mb, histopathological damage, apoptosis, inflammatory cytokines, and pyroptosis-related proteins. IMRC-Exo significantly reduced these indicators, mitigating muscle injury and inflammation. Additionally, inflammatory cytokines and pyroptosis markers were significantly lower in the IMRC-Exo group than in the Model group.

**Conclusion:**

IMRC-Exo effectively alleviates *D. acutus* venom-induced limb injury in rabbits, likely through inhibition of GSDME-dependent pyroptosis-mediated inflammation. These findings suggest that IMRC-Exo may serve as a promising therapeutic approach for snakebite-induced inflammatory injury.

## Background

Snakebite envenomation is one of the most prevalent and severe forms of animal-related injuries worldwide. According to the World Health Organization (WHO), approximately 2.7 million cases of venomous snakebites occur annually, resulting in over 100,000 deaths and 300,000 permanent disabilities [1]. In China alone, there are about 300,000 cases of snakebites each year, with fatality and disability rates reaching as high as 10% and 30%, respectively [2,3]. *Deinagkistrodon acutus*, a highly venomous snake species commonly found in China, produces hemotoxic and cytotoxic venom that is typically introduced into the bloodstream through limb bites. This venom causes severe local swelling, necrosis, and significant impairment of limb function, substantially reducing patients' quality of life. Current treatments for local wound management following *Deinagkistrodon acutus* bites include local debridement combined with vacuum sealing drainage (VSD) [4], hyperbaric oxygen therapy [5], and traditional Chinese medicine (TCM) topical applications [6]. However, these interventions are primarily applied post-injury, often with limited effectiveness, leading to delayed wound healing, secondary infections, and functional impairments. Therefore, there is an urgent need to explore effective therapeutic strategies for managing local limb injuries caused by *Deinagkistrodon acutus* bites.

Inflammation has been identified as a primary mechanism underlying snakebite-induced limb injury [7]. However, the key regulatory mechanisms and intervention strategies remain to be elucidated. Recent studies have highlighted pyroptosis, a novel form of programmed cell death [8], as a critical process that amplifies inflammatory responses, exacerbating muscle tissue damage caused by various factors [9-11]. GSDME, a key effector protein of pyroptosis, is cleaved by activated caspase-3 to release its N-terminal fragment, which inserts into the plasma membrane to form pores. This process leads to the leakage of cellular contents and triggers a robust inflammatory response [12-14]. Nevertheless, whether the GSDME-dependent pyroptosis pathway contributes to the inflammatory damage in snakebite-induced limb injuries has not yet been investigated.

Mesenchymal stem cell-derived exosomes (MSC-Exo) have been shown to exert protective effects, including anti-inflammatory, immunomodulatory, and pro-regenerative properties, in the treatment of various diseases [15, 16]. Notably, MSC-Exo has demonstrated significant efficacy in alleviating inflammatory injuries in wounds caused by diabetes and burns [17, 18]. Furthermore, immune and matrix-regulatory cells (IMRCs) differentiated from human embryonic stem cells exhibit superior cell quality - characterized by uniform morphology, stable proliferative capacity, and consistent expression of mesenchymal surface markers - as well as enhanced immunoregulatory functions and reparative capacity compared to traditional MSCs [19-21]. IMRC-Exo may therefore possess enhanced tissue-protective properties. However, the therapeutic potential and underlying mechanisms of MSC-Exo, including IMRC-Exo, in snakebite-induced wound management remain unexplored.

In this study, we aimed to establish a rabbit model of *Deinagkistrodon acutus* snakebite to confirm the occurrence of pyroptosis in limb muscle tissue following envenomation and to investigate the therapeutic potential and mechanisms of IMRC-Exo in mitigating this type of injury. We hypothesized that GSDME activation mediates pyroptosis in limb muscle tissues following snakebite and that IMRC-Exo could attenuate muscle damage by inhibiting GSDME-dependent pyroptosis and associated inflammatory responses.

## Methods

### Experimental animals

Eighteen healthy male New Zealand white rabbits (5-7 months old), with an average weight of 3.0 ± 0.1 kg, were purchased from the Fuyang Hongfeng Rabbit Breeding Farm in Hangzhou, China. All experimental procedures were performed in compliance with the guidelines of the Institutional Animal Care and Use Committee. The rabbits were housed under standard conditions: temperature controlled at 20°C to 25°C, humidity maintained at 60% to 80%, with a 12-hour light/dark cycle. They were provided with free access to water and standard rabbit feed, and the housing environment was regularly disinfected. This study was approved by the Animal Ethics Committee of Lishui University (approval no. 2023YD0113).

### Animal preparation

Before the experiment, the rabbits were acclimated under standard conditions for one week. On the day of the experiment, both hind limbs were shaved using a professional hair clipper to fully expose the limb areas. The right ear was marked for identification. Body weight was measured using a scale, and body temperature was recorded with a thermometer. Electrocardiographic monitoring (iM60, Shenzhen Mindray Bio-Medical Electronics Co., Ltd.) was connected to monitor and record baseline heart rate and oxygen saturation. Rabbits were anesthetized via intravenous injection of 3% pentobarbital solution (30 mg/kg) into the marginal ear vein.

### Randomization and interventions

**Figure 1.**
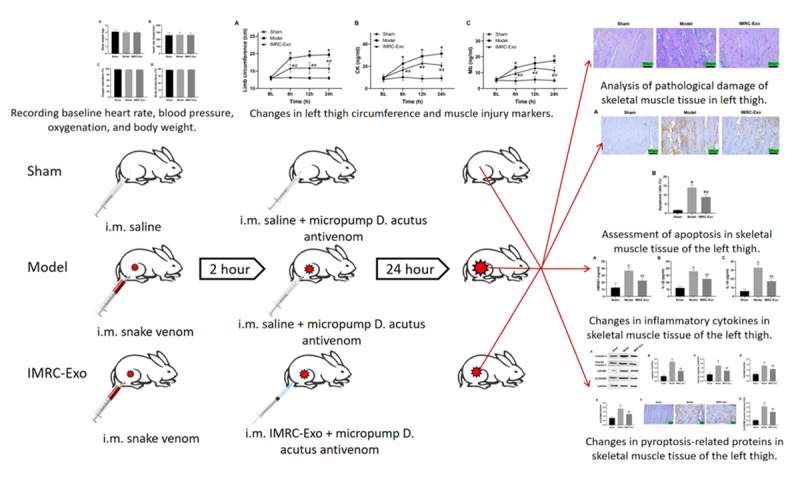
Experimental design and outcome evaluation. The red syringe represents snake venom injection, the transparent syringe indicates normal saline injection, and the blue syringe represents IMRC-Exo injection. IMRC-Exo: immune and matrix-regulatory cell-derived exosomes.

The experimental protocol and grouping strategy are illustrated in Figure 1. After animal preparation, rabbits were randomly assigned into three groups using the sealed envelope method: Sham group, Model group, and IMRC-Exo group (n = 6 per group). In the Sham group, animals underwent anesthesia and catheter placement, but no venom or therapeutic intervention was administered. In the Model group, a rabbit model of *Deinagkistrodon acutus* envenomation-induced limb injury was established by intramuscular injection of venom. The modeling procedure was adapted and optimized based on a previously established swine model of *Deinagkistrodon acutus* envenomation developed by Lai et al. [22].Two hours after venom injection, animals in the IMRC-Exo group received IMRC-Exo therapy (7.5 × 10^10^ particles, purchased from Hangzhou Luyuan Biotechnology Co., Ltd.), dissolved in 1 mL of saline and subcutaneously injected at ten sites around the venom injection point. These included four sites located 0.5 cm from the center at 0, 6, 12, and 18 o’clock positions, and six sites located 1.0 cm from the center at 0, 4, 8, 12, 16, and 20 o’clock positions, with 7.5 × 10⁹ particles per site. Sham and Model groups received equal volumes of saline administered in the same manner.

### Model establishment

Based on preliminary experiments and with reference to a previously established swine model of *Deinagkistrodon acutus* envenomation developed by Lai et al. [22], the venom concentration for modeling was set at 1.5 mg/kg. Lyophilized *Deinagkistrodon acutus* venom powder (batch number 20200410) was purchased from Shanghai Seron Biotechnology Co., Ltd., and dissolved in saline to a concentration of 100 mg/mL prior to use. The venom dose was calculated based on the body weight of each rabbit and injected perpendicularly into the mid-lateral side of the left thigh at a depth of 5 mm. The injection site was pressed with a cotton swab for 1 minute to prevent leakage. Two hours post-injection, an intravenous catheter was established in the auricular vein, and 80 U/kg of *D. acutus* antivenom diluted in 20 mL of saline (12 U/mL) was administered via an intravenous micropump. All rabbits were monitored for 6 hours post-venom injection and then returned to their cages for an 18-hour observation period. At the endpoint, rabbits were euthanized via intravenous injection of sodium pentobarbital (150 mg/kg). No unexpected adverse events were observed during the experiment. All animals tolerated the procedures well without signs of severe distress, pain, or mortality. Animals were monitored every 6 hours for signs of distress, including extreme lethargy, respiratory distress, or severe pain. No animals exhibited severe distress or required early euthanasia, and humane endpoints were not needed.

### Observational parameters

Before venom injection, the body weight and baseline vital signs (including heart rate, body temperature, and oxygen saturation) of each rabbit were recorded. Changes in the circumference of the left thigh were measured at baseline and 6, 12, and 24 hours after venom injection. At the same time point, 2 mL of venous blood was collected and centrifuged at 3000 ×g (relative centrifugal force, RCF) for 10 minutes to obtain the supernatant, which was stored at −80°C for subsequent analysis. The levels of CK and Mb were measured using enzyme-linked immunosorbent assay (ELISA) kits (CK: P0183, Myo: P0017; Shanghai Meixuan Biotechnology Co., Ltd., China) according to the manufacturer’s instructions. Specifically, 100 μL of diluted serum samples was added to the enzyme-linked immunosorbent assay wells pre-coated with the corresponding antibodies. After incubation at 37°C for 1 hour, the plates were washed three times, followed by the addition of 100 μL of enzyme conjugate for a 30-minute incubation. The plates were washed again, and the substrate solution was added for color development. The optical density (OD) was read at 450 nm, and the concentrations of CK and Mb were calculated using standard curves.

At the experimental endpoint, muscle tissue samples were collected 2 cm below the venom injection site and fixed in 10% neutral formalin for 24 hours. The tissues were then processed with routine dehydration, clearing, and paraffin embedding, followed by slicing into 4-μm-thick sections. Histopathological damage was assessed using Hematoxylin and Eosin (HE) staining. Briefly, sections were stained with hematoxylin for 5 minutes, rinsed with tap water, counterstained with eosin for 1 minute, and then dehydrated, cleared, and mounted. A light microscope (Olympus, Tokyo, Japan) at 200× magnification was used to observe three randomly selected fields for signs of muscle fiber necrosis and inflammatory cell infiltration. Additionally, apoptosis in muscle tissues was evaluated using Terminal Deoxynucleotidyl Transferase dUTP Nick-End Labeling (TUNEL) staining. Tissue sections were pretreated with proteinase K and stained using a TUNEL kit (Wuhan Boster Biological Technology Co., Ltd., China) following the manufacturer’s instructions. Under a biological microscope (C×31, Olympus, Japan) at 200× magnification, three random fields were photographed, and the ratio of TUNEL-positive cells to total cells was calculated as the apoptosis rate. The expression of N-GSDME in muscle was measured by immunohistochemistry at 24 h after the model establishment. Similarly, muscle tissue samples were obtained, then fixed and embedded, and finally sliced into 4-μm-thick sections. The sections were incubated with primary anti-N-GSDME (1:200, Cell Signaling Technology, Danvers, United States), then treated with the secondary antibody, and finally reacted with diaminobenzidine (Boster Biological Technology, Wuhan, China). Three fields were randomly photographed at 200× magnification with the CX31 optical microscope. The semiquantitative analysis of the intensity of N-GSDME positive staining were performed through integrated optical density (IOD) using the Image-Pro Plus 6.0 software (Media Cybernetics, Silver Spring, United States).

At the experimental endpoint, muscle tissue samples were collected as described above, immediately flash-frozen in liquid nitrogen, and stored at −80°C for subsequent analyses. During the experiments, the frozen samples were thawed and homogenized. The concentrations of HMGB1, IL-1β, and IL-18 were measured using ELISA kits (IL-1β: P0002, IL-18: P0002; Shanghai Meixuan Biotechnology Co., Ltd., China) according to the manufacturer’s instructions. Briefly, 100 μL of tissue homogenate was added to enzyme-linked immunosorbent assay wells pre-coated with specific antibodies and incubated at 37°C for 1 hour. The plates were washed three times, followed by the addition of 100 μL of enzyme conjugate for a 30-minute incubation. After washing, substrate solution was added and incubated for 15 minutes. The reaction was terminated by adding 50 μL of stop solution, and the OD was measured at 450 nm. The concentrations of HMGB1, IL-1β, and IL-18 were calculated using standard curves. The supernatants of muscle tissue homogenates were also used for western blotting of protein concentrations of caspase-3, cleaved caspase-3, GSDME, and N-GSDME. As follows, the samples were separated by SDS- PAGE, then transferred to a polyvinylidene fluoride membrane, and finally blocked with 5% nonfat milk. Subsequently, the membranes were incubated with primary antibodies to caspase-3 (1:1,000; Proteintech, Rosemont, IL, USA), cleaved caspase-3 (Cell Signaling Technology, Danvers, MA, USA), GSDME (1:1,000; Proteintech, Rosemont, IL, USA), N-GSDME (1:1,000; Abcam, Cambridge, UK), and glyceraldehyde 3-phosphate dehydrogenase (GAPDH, 1:1,000; BBI Life Sciences Corporation, Shanghai, China). Thereafter, anti-mouse antibody (1:5,000; BBI Life Sciences Corporation, Shanghai, China) or anti-rabbit antibody (1:5,000; BBI Life Sciences Corporation, Shanghai, China) was used as the secondary antibody. Finally, the band intensities were quantified using Image J software (National Institutes of Health, Bethesda, MD, USA), and the results were all standardized by GAPDH.

### Statistical analysis

Statistical analysis was performed using Statistical Package for the Social Sciences (SPSS) 20.0 software (IBM Corporation, United States). The normality of the data was assessed using the Kolmogorov-Smirnov test. Data following a normal distribution were expressed as the mean ± standard deviation (± SD). Intergroup comparisons were conducted using one-way ANOVA, and post hoc pairwise comparisons were performed with the Bonferroni test. A p-value < 0.05 was considered statistically significant.

## Results

### Survival and baseline characteristics

**Figure 2.**
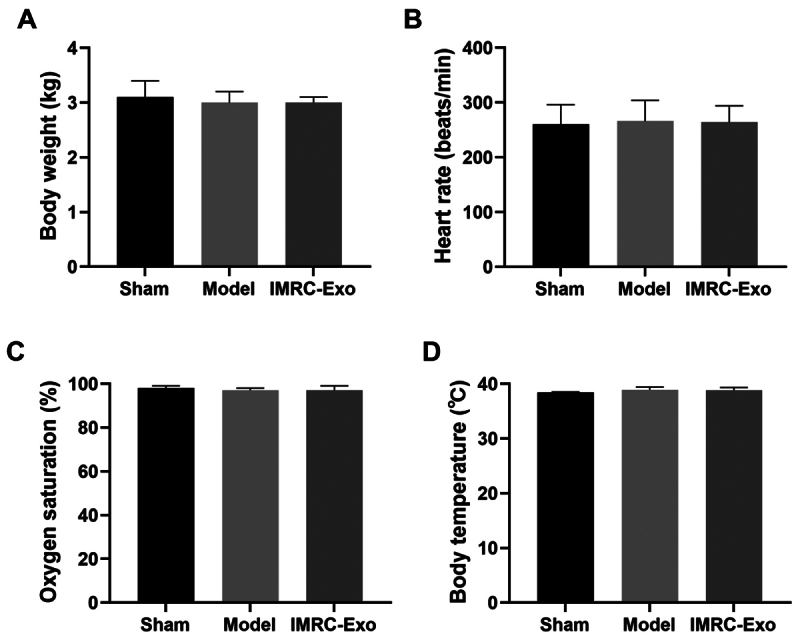
Initial body weight and baseline vital signs. **(A)** Initial body weight; **(B)** heart rate; **(C)** oxygen saturation; **(D)** body temperature. IMRC-Exo: immune and matrix-regulatory cell-derived exosomes.

A total of 18 rabbits were included in this study, and all animals survived the 24-hour observation period. Before venom injection, baseline characteristics, including body weight, heart rate, body temperature, and oxygen saturation, were within normal ranges for all groups, with no significant differences between groups (*p* > 0.05) (Figure 2).

### Changes in left thigh circumference and serum CK and Mb levels

**Figure 3.**
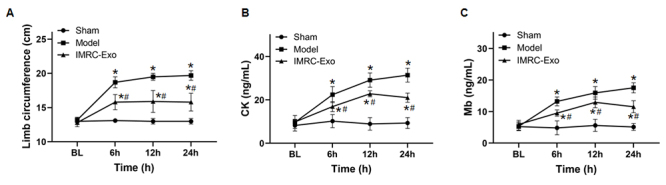
Changes in left thigh circumference and muscle injury markers. **(A)** Left thigh circumference; **(B)** creatine kinase (CK); **(C)** myoglobin (Mb). *p ( 0.05 compared to the Sham group; #*p* ( 0.05 compared to the model group. BL: baseline; IMRC-Exo: immune and matrix-regulatory cell-derived exosomes.

Before venom injection, there were no significant differences in left thigh circumference or serum CK and Mb concentrations between the three groups (*p* > 0.05). After venom injection, both the Model and IMRC-Exo groups exhibited significant increases in left thigh circumference and serum CK and Mb levels compared with the Sham group. However, the IMRC-Exo group showed significantly reduced left thigh circumference and serum CK and Mb concentrations compared to the Model group (*p* < 0.05) (Figure 3).

### Histopathological assessment of muscle tissue

**Figure 4.**
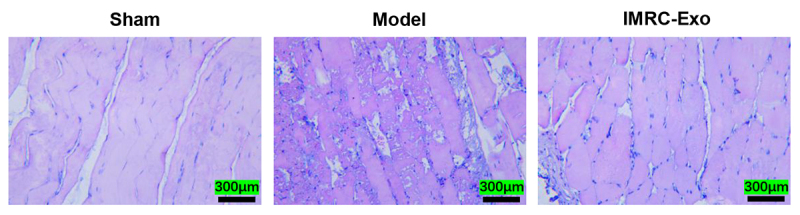
Analysis of pathological damage of skeletal muscle tissue in left thigh (representative micrographs of HE staining, × 200 magnification). IMRC-Exo: immune and matrix-regulatory cell-derived exosomes.

At 24 hours post-venom injection, HE staining in the Sham group revealed normal muscle tissue structure. In contrast, the Model group exhibited severely disorganized muscle architecture, characterized by muscle fiber rupture and necrosis, accompanied by inflammatory cell infiltration and interstitial edema. Compared to the Model group, the IMRC-Exo group demonstrated more intact muscle structure, with orderly fiber arrangement, reduced rupture and necrosis, and significantly alleviated inflammatory cell infiltration and edema (Figure 4).

### Apoptosis in muscle tissue

**Figure 5.**
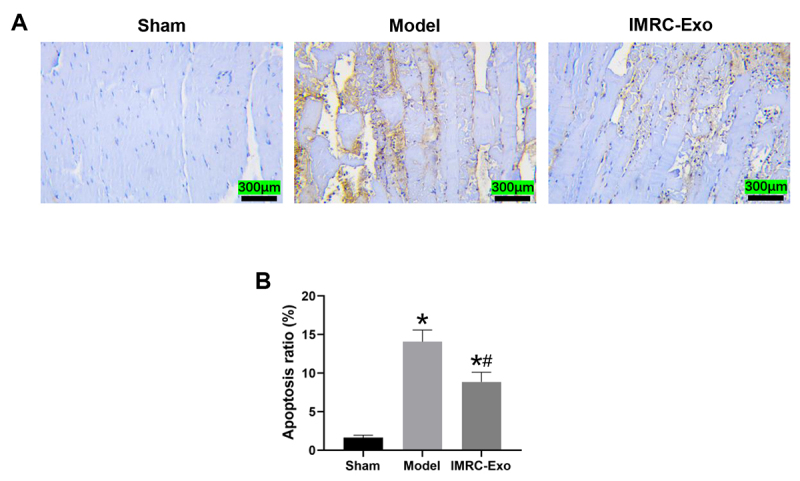
Assessment of apoptosis in skeletal muscle tissue of the left thigh. **(A)** Representative images of TUNEL staining (200× magnification). **(B)** Analysis of apoptosis rates. *p ( 0.05 compared to the Sham group; #*p* ( 0.05 compared to the Model group. IMRC-Exo: immune and matrix-regulatory cell-derived exosomes.

At 24 hours post-venom injection, minimal apoptosis was observed in the Sham group. As shown in Figure 5A, the Model group exhibited a marked increase in apoptotic cells, evidenced by a substantial number of brownish yellow TUNEL-positive cells. However, IMRC-Exo intervention significantly reduced the number of apoptotic cells. Quantitative analysis of the apoptosis rate (Figure 5B) indicated that apoptosis in muscle tissues was significantly increased in both the Model and IMRC-Exo groups compared with the Sham group (*p* < 0.05). Nevertheless, the apoptosis rate was significantly lower in the IMRC-Exo group than in the Model group (*p* < 0.05).

### Inflammatory cytokine levels in muscle tissue

**Figure 6.**
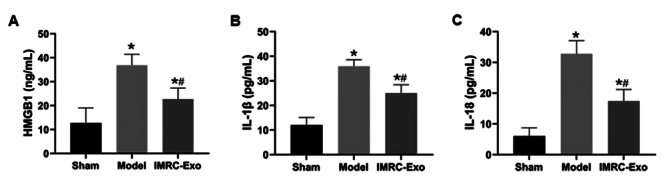
Changes in inflammatory cytokines in skeletal muscle tissue of the left thigh. **(A-C)** ELISA analysis of high mobility group box 1 (HMGB1), interleukin-1β (IL-1β), and interleukin-18 (IL-18) levels. *p ( 0.05 compared to the Sham group; #*p* ( 0.05 compared to the Model group. IMRC-Exo: immune and matrix-regulatory cell-derived exosomes; IMRC-Exo: immune and matrix-regulatory cell-derived exosomes.

At 24 hours post-venom injection, ELISA results showed that the concentrations of HMGB1, IL-1β, and IL-18 in muscle tissue were significantly elevated in both the Model and IMRC-Exo groups compared with the Sham group (*p* < 0.05). However, these inflammatory cytokine levels were significantly lower in the IMRC-Exo group compared with the Model group (*p* < 0.05) (Figure 6).

### Pyroptosis-related protein expression

**Figure 7.**
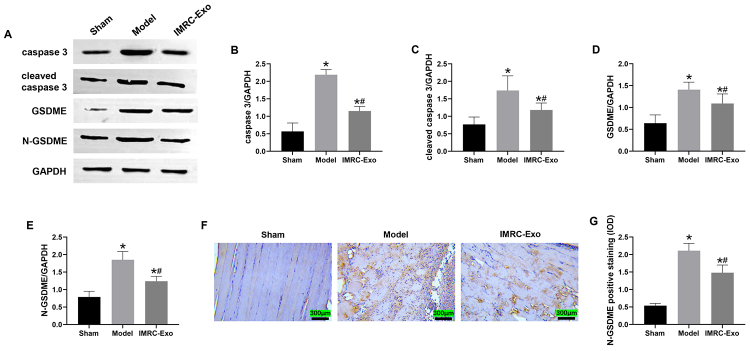
Changes in pyroptosis-related proteins in skeletal muscle tissue of the left thigh. **(A)** Western blot analysis of caspase-3, cleaved caspase-3, gasdermin E (GSDME), and N-terminal GSDME (N-GSDME) protein bands. **(B-E)** Quantitative analysis of caspase-3, cleaved caspase-3, GSDME, and N-GSDME expression levels. Band intensities were measured using ImageJ software (NIH, USA) and normalized to β-actin. The Y-axis indicates the relative protein band intensity. **(F)** Representative immunohistochemical staining images of N-GSDME (200× magnification). **(G)** Analysis of IOD values for N-GSDME-positive staining. *p < 0.05 compared to the Sham group; #*p* < 0.05 compared to the Model group. Full-length, uncropped Western blot images corresponding to panel (A) are provided in Additional file 1, including original blots for GSDME, N-GSDME, caspase-3, cleaved caspase-3, and GAPDH. IMRC-Exo: immune and matrix-regulatory cell-derived exosomes.

Western blot analysis at 24 hours post-venom injection revealed significantly increased expression of caspase-3, cleaved caspase-3, GSDME, and N-GSDME in the Model and IMRC-Exo groups compared with the Sham group (*p* < 0.05). However, the IMRC-Exo group exhibited significantly lower expression levels of these pyroptosis-related proteins compared with the Model group (*p* < 0.05). Furthermore, immunohistochemical analysis demonstrated significantly higher IOD values of N-GSDME-positive staining in muscle tissues from the Model and IMRC-Exo groups compared with the Sham group. Notably, IMRC-Exo treatment significantly reduced N-GSDME-positive IOD values compared with the Model group (*p* < 0 .05) (Figure 7).

## Discussion

In this study, we observed the occurrence of pyroptosis in limb muscle tissues of rabbits following *Deinagkistrodon acutus* snakebite and evaluated the protective effects and underlying mechanisms of IMRC-Exo in mitigating snakebite-induced limb injury. Our results demonstrated that IMRC-Exo significantly reduced limb swelling, decreased levels of muscle injury biomarkers, and alleviated histopathological damage and apoptosis in muscle tissues, thereby exerting a protective effect. Furthermore, we found that GSDME activation and GSDME-mediated pyroptosis occurred in limb muscle tissues following snakebite, but IMRC-Exo markedly inhibited this process and reduced inflammatory damage, which may underlie its protective effects.

MSC-Exo are membrane-bound nanovesicles enriched with proteins, nucleic acids, and lipids, and exhibit various biological effects, including anti-inflammatory, antioxidant, and pro-angiogenic activities. These properties enable MSC-Exo to protect cells and promote tissue repair in diverse settings [23]. Previous studies have shown that MSCs and their soluble components significantly attenuate ischemic injury in limb muscles through anti-inflammatory and regenerative mechanisms, highlighting their potential for muscle protection and repair [24]. In particular, MSC-Exo have been shown to inhibit pyroptosis via the circHIPK3/FOXO3a pathway, thereby promoting muscle repair and regeneration and effectively alleviating ischemic injury [25]. Additionally, MSC-Exo have demonstrated therapeutic potential in wound healing across various injury models. For instance, Yang J et al. [26] found that umbilical cord-derived MSC-Exo combined with hydrogels enhanced wound healing and skin regeneration in diabetic rats. Similarly, Li et al. [27] reported that MSC-Exo accelerated wound healing in a diabetic foot ulcer mouse model by promoting fibroblast proliferation and migration while inhibiting apoptosis and inflammation. Moreover, Bo et al. [28] demonstrated that pluripotent stem cell-derived exosomes promoted keratinocyte and endothelial cell migration through miR-762, thereby expediting burn wound repair. Recent findings also suggest that other immune cells, such as dendritic cells (DCs), may contribute to muscle regeneration after envenomation. Silva et al. demonstrated that bone marrow-derived DCs attenuated local inflammation and facilitated the onset of tissue repair following Bothrops jararacussu venom injury by reducing cytokine levels and histological damage [29]. However, the application of MSC-Exo for snakebite-induced limb injury has not yet been investigated.

Based on the above evidence, this study aimed to explore the effects of MSC-Exo on snakebite-induced limb injury. We specifically utilized exosomes derived from IMRCs, as IMRCs - originating from human embryonic stem cells - have been shown to outperform MSCs from adult tissues in terms of anti-inflammatory and immunomodulatory properties [30, 31]. To ensure clinical relevance, we referenced the venom dosage used in previous *Deinagkistrodon acutus* pig models [22] and determined an intramuscular injection dose of 1.5 mg/kg based on our preliminary experiments. Venom was injected into the mid-lateral side of the left thigh, a common site of snakebites in clinical settings, to mimic the pathological changes observed in humans. The venom injection resulted in significant limb swelling, elevated muscle injury biomarkers, histopathological damage, and apoptosis in muscle tissues, confirming successful model establishment. Notably, IMRC-Exo treatment significantly mitigated these abnormalities, demonstrating its protective effects against snakebite-induced limb injury.

Pyroptosis has been implicated in the pathophysiological processes of various wounds and has emerged as a promising therapeutic target. Wang et al. [32] demonstrated that inhibiting pyroptosis activation promoted wound healing in diabetic foot ulcers. Similarly, Hasan Maleki et al. [33] found that nicotinamide riboside and resveratrol aided in diabetic wound repair by suppressing pyroptosis. Zhang et al. [34] reported that bioactive glass accelerated wound healing in soft tissue injuries by modulating connexin 43/reactive oxygen species signaling to inhibit endothelial cell pyroptosis. In addition, recent studies have shown that snake venom components may activate the NLRP3 inflammasome and GSDMD-mediated pyroptosis pathways in immune cells such as macrophages, contributing to local inflammation and muscle damage [35-37]. These findings highlight the importance of exploring alternative pyroptosis pathways, including GSDME-mediated pyroptosis, in venom-induced injury models. Furthermore, MSC-Exo have been shown to regulate pyroptosis and confer protection in various organ injury models. For example, Liu et al. [38] demonstrated that MSC-Exo alleviated LPS-induced acute lung injury by inhibiting alveolar macrophage pyroptosis. Additionally, adipose-derived MSC-Exo significantly improved liver ischemia-reperfusion injury by suppressing pyroptosis and apoptosis-related pathways, reducing systemic inflammation and liver tissue damage [39]. Exosomes from M2-polarized microglia (M2-Exos) have also been found to inhibit neuronal pyroptosis via ubiquitin-mediated degradation of TXNIP, thereby mitigating cerebral ischemia-reperfusion injury [40].

In this study, we further investigated the role of pyroptosis in snakebite-induced muscle injury and the potential mechanisms underlying the protective effects of IMRC-Exo. Our results revealed that venom injection led to significant increases in the expression of pyroptosis-related proteins, including caspase-3, cleaved caspase-3, GSDME, and N-GSDME, as well as elevated levels of inflammatory cytokines HMGB1, IL-1β, and IL-18. These findings indicate the involvement of GSDME-mediated pyroptosis in snakebite-induced muscle injury. Importantly, IMRC-Exo treatment significantly reduced the expression of these pyroptosis-related proteins and inflammatory cytokines, suggesting that IMRC-Exo attenuated inflammatory injury by inhibiting GSDME-mediated pyroptosis.

This study has several limitations. First, the observation period was relatively short, which may not fully capture the dynamic changes in limb injury following snakebite. Future studies should extend the observation period to comprehensively evaluate the progression of injury and the therapeutic effects of IMRC-Exo. Second, we only used a single dose of IMRC-Exo and did not explore dose-dependent effects on treatment outcomes. Dose optimization in future studies could help identify the most effective therapeutic regimen. Finally, while this study focused on key markers of the GSDME pyroptosis pathway, further investigations are needed to elucidate the molecular mechanisms through which IMRC-Exo regulate pyroptosis in snakebite models. Notably, a parallel group treated with a pyroptosis inhibitor was not included in this study, which limits the strength of mechanistic validation.

## Conclusion

This study provides the first evidence that IMRC-Exo exerts protective effects against limb wound damage induced by *Deinagkistrodon acutus* snakebite by inhibiting GSDME-mediated pyroptosis and the associated inflammatory injury. Our findings offer new perspectives and potential therapeutic strategies for the clinical management of snakebite-induced wounds.

## Abbreviations

CK: creatine kinase; DCs: dendritic cells; ELISA: enzyme-linked immunosorbent assay; Exo: exosomes; GSDME: gasdermin E; HE: hematoxylin and eosin; i.m.: intramuscular; IMRC: immune and matrix-regulatory cells; IOD: integrated optical density; Mb: myoglobin; N-GSDME: N-terminal gasdermin E; OD: optical density; SPSS: statistical package for the social sciences; TCM: traditional Chinese medicine; TUNEL: transferase dUTP nick-end labeling; VSD: vacuum sealing drainage; WHO: World Health Organization.

## Data Availability

The datasets generated and/or analyzed during the current study are available from the corresponding author upon reasonable request.

## Supplementary material

The following online material is available for this article:

Additional file 1. Uncropped blots for caspase-3，cleaved caspase-3, GAPDH, GSDME, and N-GSDME used in Figure 7A. Sample lanes from left to right: lane 7, Sham; lane 8, Model; lane 9, IMRC-Exo.
