# Supplementary material for: IMRC-Exo mitigates Deinagkistrodon acutus venom-induced limb injury in rabbits by inhibiting GSDME-dependent pyroptosis
Source: J Venom Anim Toxins Incl Trop Dis. 2025 Sep 5;31:e20230009. doi: 10.1590/1678-9199-JVATITD-2025-0009 (PMC12412906; doi:10.1590/1678-9199-JVATITD-2025-0009)
Supplement: Additional file 1. [file 1678-9199-jvatitd-31-e20230009-s1.pdf]

J Venom Anim Toxins incl Trop Dis
https://doi.org/10.1590/1678-9199-JVATITD-2025-0009

Supplementary Material to "IMRC-Exo mitigates *Deinagkistrodon acutus* venom - induced limb injury in rabbits by inhibiting GSDME-dependent pyroptosis"

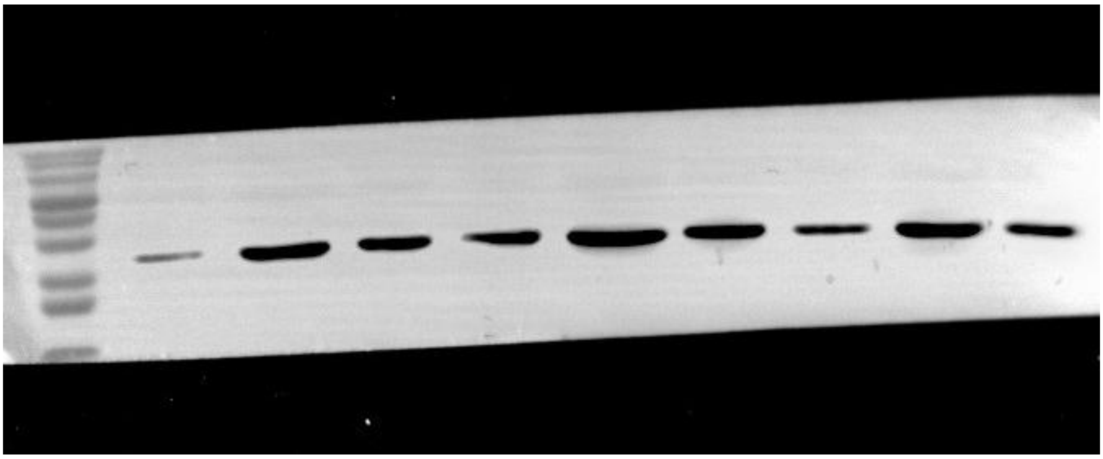

Caspase-3

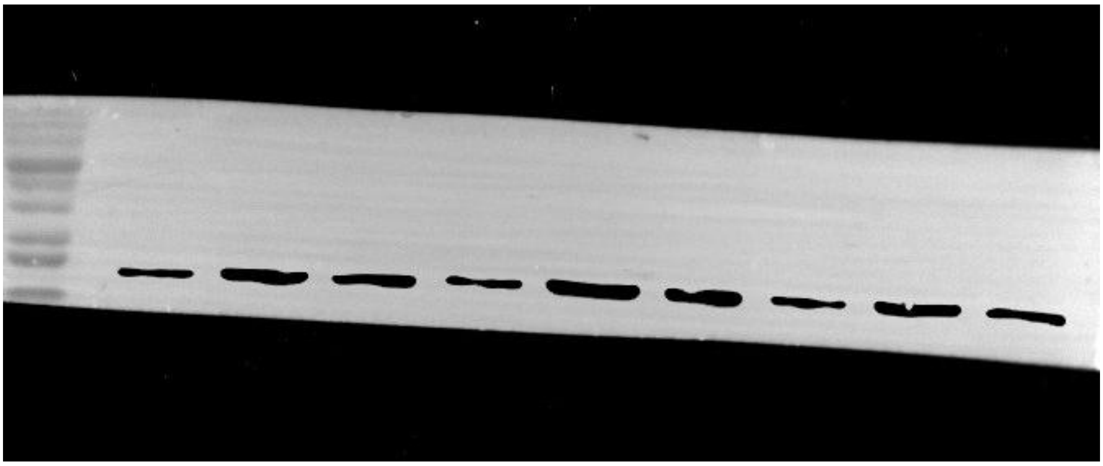

Cleaved caspase-3

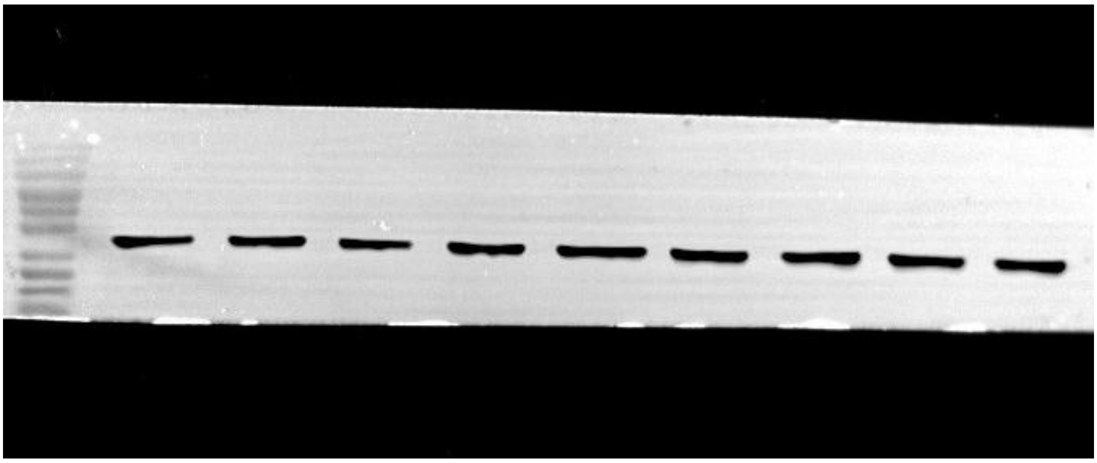

GAPDH

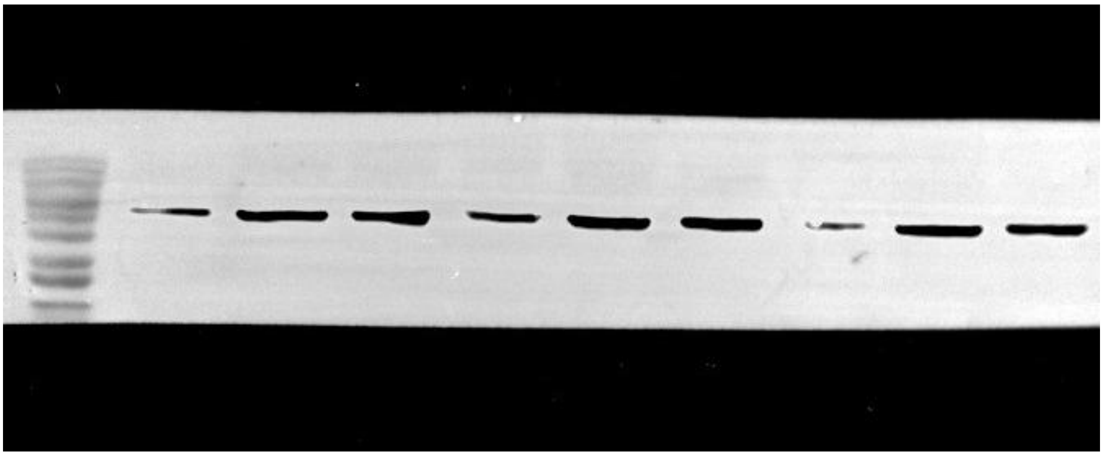

GSDME

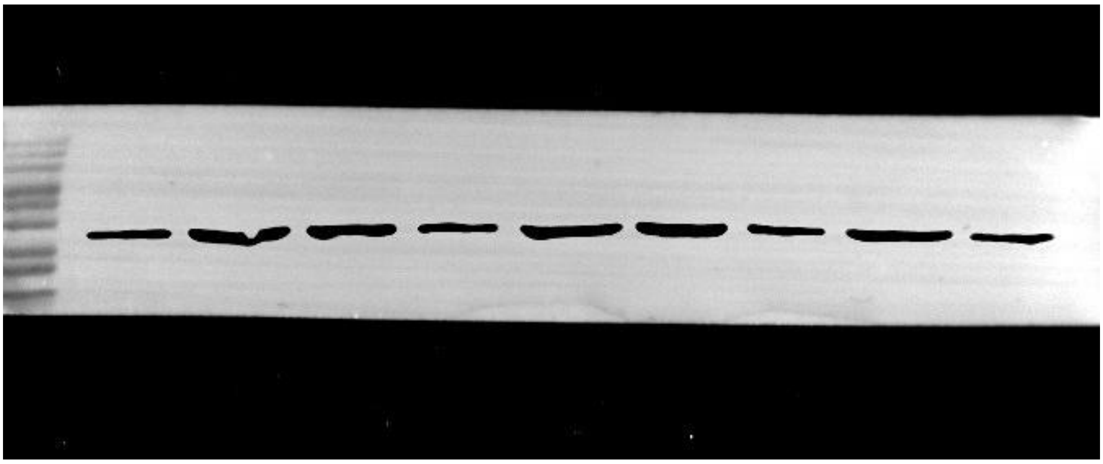

N-GSDME

Additional file 1. Uncropped blots for caspase-3， cleaved caspase-3, GAPDH, GSDME, and N-GSDME used in Figure 7A. Sample lanes from left to right: lane 7, Sham; lane 8, Model; lane 9, IMRC-Exo.
